# ALLENE OXIDE SYNTHASE and HYDROPEROXIDE LYASE, Two Non-Canonical Cytochrome P450s in *Arabidopsis thaliana* and Their Different Roles in Plant Defense

**DOI:** 10.3390/ijms20123064

**Published:** 2019-06-23

**Authors:** Sachin Rustgi, Armin Springer, ChulHee Kang, Diter von Wettstein, Christiane Reinbothe, Steffen Reinbothe, Stephan Pollmann

**Affiliations:** 1 Department of Plant and Environmental Sciences, Pee Dee Research and Education Center, Clemson University, Florence, SC 29506, USA; srustgi@clemson.edu; 2 Department of Crop and Soil Sciences, Washington State University, Pullman, WA 99164, USA; diter@wsu.edu; 3 Medizinische Biologie und Elektronenmikroskopisches Zentrum (EMZ), Universitätsmedizin Rostock, 18055 Rostock, Germany; Armin.Springer@med.uni-rostock.de; 4 Department of Chemistry, Biomolecular Crystallography Center, Washington State University, Pullman, WA 99164, USA; chkang@wsu.edu; 5 Biologie Environnementale et Systémique (BEEeSy), Université Grenoble Alpes, BP 53, CEDEX, F-38041 Grenoble, France; bt434375@uni-bayreuth.de; 6 Centro de Biotecnología y Genómica de Plantas, Universidad Politécnica de Madrid (UPM)—Instituto Nacional de Investigación y Tecnología Agraria y Alimentación (INIA), Campus de Montegancedo, 28223 Pozuelo de Alarcón, Madrid, Spain

**Keywords:** allene oxide synthase, allene oxide cyclase, chloroplast envelope protein complex, hydroperoxide lyase, lipoxygenase, metabolite channeling, plant defense

## Abstract

The channeling of metabolites is an essential step of metabolic regulation in all living organisms. Multifunctional enzymes with defined domains for metabolite compartmentalization are rare, but in many cases, larger assemblies forming multimeric protein complexes operate in defined metabolic shunts. In *Arabidopsis thaliana*, a multimeric complex was discovered that contains a 13-lipoxygenase and allene oxide synthase (AOS) as well as allene oxide cyclase. All three plant enzymes are localized in chloroplasts, contributing to the biosynthesis of jasmonic acid (JA). JA and its derivatives act as ubiquitous plant defense regulators in responses to both biotic and abiotic stresses. AOS belongs to the superfamily of cytochrome P450 enzymes and is named CYP74A. Another CYP450 in chloroplasts, hydroperoxide lyase (HPL, CYP74B), competes with AOS for the common substrate. The products of the HPL reaction are green leaf volatiles that are involved in the deterrence of insect pests. Both enzymes represent non-canonical CYP450 family members, as they do not depend on O_2_ and NADPH-dependent CYP450 reductase activities. AOS and HPL activities are crucial for plants to respond to different biotic foes. In this mini-review, we aim to summarize how plants make use of the LOX2–AOS–AOC2 complex in chloroplasts to boost JA biosynthesis over volatile production and how this situation may change in plant communities during mass ingestion by insect pests.

## 1. Introduction

Oxygenated membrane fatty acid derivatives such as lipoxins, leukotrienes, thromboxanes, and prostaglandins are widespread in occurrence in metazoans and play important roles in many physiological processes. Often, collectively referred to as eicosanoids, lipoxins, leukotrienes, thromboxanes, and prostaglandins accomplish key functions in inflammatory processes (lipoxins and leukotrienes), host defense against pathogens, vasoconstriction and vasodilatation, muscle contraction, blood pressure regulation, blood coagulation (prostaglandins and thromboxanes), and collectively act in many other processes [1,2,3]. Interestingly, eicosanoid-like compounds have been identified in soft corals and other marine organisms where they presumably act in the deterrence of microbial pathogens [4,5,6].

**Figure 1:**
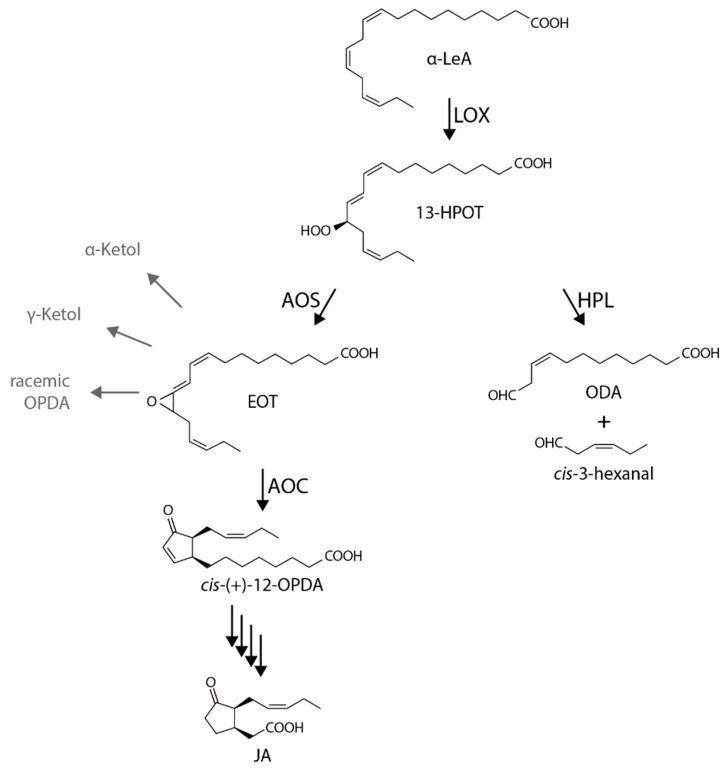
Biosynthesis of jasmonic acid (JA) and green leaf volatiles through the Vick and Zimmerman shunt. Pathway intermediates are given as 13-HPOT, (9*Z*11*E*15*Z*13*S*)-13-hydroperoxy-9,11,15-octadecatrienoic acid; α-LeA, α-linolenic acid; EOT, 12,13(*S*)-epoxy-9(*Z*),11,15(*Z*)-octadecatrienoic acid; OPDA, *cis*-(+)-12-oxophytodienoic acid; ODA, 12-oxo-*cis*-9-dodecenoic acid. Enzymes abbreviations refer to LOX, 13-lipoxygenase; AOS, 13-allene oxide synthase; AOC, allene oxide cyclase; HPL, 13-hydroperoxide lyase. Note that 13-HPOT is a common substrate of AOS and HPL that, as non-canonical CYP450 enzymes, drive alternative reactions in the JA and green leaf volatile branches of the Vick and Zimmerman pathway.

What appears to be prostaglandins in animals are jasmonates in plants (Figure 1). Jasmonates regulate flower development, embryogenesis, seed germination, and fruit ripening [7]. Jasmonic acid (JA) accumulates in response to both abiotic and biotic stresses and is additionally involved in wound responses and defense [8,9,10]. Pioneering work by Weigel and colleagues identified the overlapping roles of JA in leaf development and foliar senescence [11]. In addition, an emerging number of findings suggest complex crosstalk with other plants hormones (e.g., [12]).

When plants are challenged by biotic and abiotic foes, they synthesize defense compounds that permit plant survival. Accumulation of a wide range of defense compounds has been reported, and the key elements of JA signaling were identified [10,13]. Insect feeding provokes overlapping as well as distinct defenses that include local and systemic reactions and additionally lead to the attraction of the insects’ enemies through the emission of green leaf volatiles comprising *Z*-3-hexenal and the corresponding C6-volatile alcohol [(*Z*)-3-hexenol], thus, providing an indirect defense involving interplant communication [14,15,16,17,18,19,20,21,22,23,24].

Interestingly, both prostaglandins and JAs are lipoxygenase (LOX) pathway products, although the LOX enzymes involved have different substrate specificities and, thus, can drive the formation of different compounds [10,25,26,27]. It is also interesting to recall that both JAs and green leaf volatiles are derived from the same 13-LOX pathway and that their synthesis diverges at an early common step (Figure 1). The 13-LOX pathway originally discovered by Vick and Zimmerman [28,29] commences with the release of α-linolenic acid from membrane lipids by phospho- and galactolipases. Subsequent steps then include 13-lipoxygenase (LOX), 13-allene oxide synthase (AOS, At5g42650), and allene oxide cyclase (AOC) carrying out consecutive steps in chloroplasts (Figure 1) [30,31,32,33,34,35]. The product of the regio- and stereospecific 13-LOX (EC 1.13.11.12) reaction is (13*S*)-hydroperoxylinolenic acid (13-HPOT). This compound is, likewise, used as a substrate by AOS (EC 4.2.1.92) and 13-hydroperoxide lyase (HPL, EC 4.2.99.X) to provide different products. Whereas AOS converts 13-HPOT to 12,13-epoxylinolenic acid (EOT), HPL cleaves 13-HPOT to *Z*-3-hexenal and 12-oxo-*cis*-9-dodecenoic acid (ODA) of which *cis*-3-hexenal and the corresponding alcohol are volatile compounds operative in herbivore deterrence [21,36,37,38,39]. Here, the strict regiospecificity of 13-LOX, 13-AOS, and 13-HPL has to be highlighted, as there are also other enzymes that show a specificity for the 9-position, such as 9-HPL that belongs to the CYP74C group [30]. Because EOT as the product of the AOS reaction is short-lived and spontaneously disintegrates into volatile α- and γ-ketols as well as racemic 12-oxo-phytodienoic acid (OPDA), plants use, in terms of AOC (EC 5.3.99.6), an enzyme that assures *cis*-(+)-12-oxo-phytodienoic acid (*cis*-(+)-12-OPDA) synthesis (Figure 1). *cis*-(+)-12-OPDA formed by AOC is then exported from chloroplasts to the cytosol and, subsequently, to peroxisomes where the final reduction and β-oxidation steps of JA biosynthesis occur. The *Arabidopsis thaliana* genome initiative identified six genes encoding LOX isoforms and one and four genes encoding AOS and AOC enzymes, respectively [40]. Just one gene encodes HPL (At4g15440).

## 2. AOS and HPL Are Examples of Non-Canonical Cytochrome P450 Enzymes with Unique Activities in Chloroplasts

Both AOS and HPL belong to the same cytochrome P450 superfamily, representing non-canonical members and are therefore designated CYP74A and CYP74B, respectively. In contrast to canonical cytochrome P450s, both AOS and HPL do not require O_2_ and NADPH-dependent cytochrome P450 reductase for activity [31,41,42]. Their amino acid sequences are moderately conserved and differ mostly in their catalytic site residues while still forming a similar hydrophobic substrate binding pocket. Remarkably, other non-canonical CYP450s, such as divinyl-ether synthases (DES), which belong to the CYP74D group, have not been identified in the Arabidopsis genome [43].

Crystal structure analyses on AOS of *Arabidopsis thaliana* and guayule (*Parthenium argentatum*) [44], in combination with site-directed mutagenesis, have provided valuable insights into the substrate binding and conversion modes of AOS and HPL. According to Li et al. [45], AOS possesses the characteristic CYP450 fold, a 22-Å deep substrate access channel, as well as a unique heme-binding site. In addition, AOS exhibits a non-conventional membrane binding mode. AOS shares with mammalian CYP2C5 [46] the same macromolecular surface to interact with the membrane. This surface comprises at least two detergent-exposed α-helices [44]. AOS from guayule looks, in fact, quite similar to the AOS of Arabidopsis in terms of its overall topology and active site residues [47]. As found for other cytochrome P450 enzymes [48,49], substrate binding also confers reaction specificity to AOS and HPL, respectively. 

Upon substrate binding to AOS, the carboxyl group establishes hydrogen bonds with Thr389, whereas the aliphatic segments establish hydrophobic contacts with neighboring non-polar amino acid side chains. On the other hand, the peroxy group of the substrate approaches the co-factor heme and forms productive interactions with the catalytic Asn321 residue. The crystal structure described by Lee et al. [44] additionally identified an unusual active site poised to control the reactivity of an epoxyallylic radical and its cation by means of interactions with an aromatic pi-system of a conserved Phe residue (Phe137). As found for other CYP74 enzymes, AOS and HPL share the presence of a nine-residue insertion in the proximal Cys loop that contributes to diminishing the donor strength of the thiolate and favoring the generation of S–Fe(IV)–OH complex that, in turn, can readily participate in electron transfer (AOS) or oxygen rebound (HPL). Replacing the amino acids involved in these steps by non-polar residues, as encountered in the AOS(F137L, S155A) mutant derivative, prevents a carbocation intermediate from being adequately enriched or stabilized at C11 and prevented the formation of an unstable hemiacetal [50], which spontaneously dissociates into short-chain aldehydes. As a result, markedly reduced AOS activity was observed while converting the mutant enzyme into an HPL-like enzyme [44]. Obviously, AOS clips the C11 position of its substrate in between two pi-systems to ensure that 13(*S*)-HPOT is efficiently converted to allene oxide and not into short-chain aldehydes. On the other hand, the HPL active site evolved to facilitate radical rearrangement by excluding a strategically positioned aromatic residue (Phe137) in the vicinity of C11 of 13(*S*)-HPOT [27]. 

As said before, AOS and HPL are encoded by distinct genes in Arabidopsis. The gene At4g15440 encodes an HPL protein that is supposed to contain a 25 amino acid chloroplast transit peptide (cTP) while, according to the Subcellular Localization Database for Arabidopsis Proteins (SUBA) [51], this protein is predicted to be non-chloroplastic. HPL was experimentally proven by in vivo- and in vitro-approaches to be present in chloroplasts [52,53]. However, its predicted localization using ChloroP and TargetP provided contradictory results and suggested the presence of mitochondrial and chloroplast transit peptides, respectively [54,55]. For AOS, more clear prediction results were obtained, highlighting the presence of a 32 amino acid cTP. As shown by our recent studies [53], AOS is faithfully imported into isolated chloroplasts and localized to the inner plastid envelope where it faced the inter-membrane space separating the outer and inner envelope. HPL was readily taken up as well in the chloroplast import experiments but accumulated in the outer plastid envelope and faced the cytosol. During import, both AOS and HPL were proteolytically processed and their cTPs cleaved off/removed. Collectively, our data are consistent with previous proteomics and localization studies using green fluorescent protein (GFP) technology and unequivocally identified AOS and HPL to be chloroplast proteins [52,56].

AOS forms larger complexes with the two other entry enzymes of the 13-LOX pathway, that is, LOX2 (At3g45140) and AOC2 (At3g25770). All three enzymes, in fact, cooperate structurally and functionally to drive OPDA synthesis from α-LeA. A remarkable channeling of α-LeA towards *cis*-(+)-12-OPDA was observed that prevented the release of any of the reaction intermediates into the incubation medium. Due to the observed channeling/compartmentalization of reactants, both the spontaneous dismutation of EOT to its short-lived disintegration products (α-ketols and γ-ketols) as well as of racemic OPDA did not occur. Moreover, a large, approximately 120-fold increase in the yield of *cis*-(+)-12-OPDA from α-LeA was observed. In marked contrast to these results, we were unable to identify proteins interacting with HPL in chloroplasts in our protein isolation and crosslinking experiments. This result may suggest that HPL establishes weak interactions that could not be traced by the methods employed or that the interacting partners were not present in chloroplasts from 14 d-old light-grown, healthy plants [53].

## 3. Structural Modelling of the LOX2–AOS–AOC2 Plastid Envelope Complex

The fact that AOS forms complexes with LOX2 and AOC2 encouraged us to perform a molecular modeling of the whole complex. An interesting precedent for the interaction of enzymes involved in oxylipin biosynthesis was provided by the soft coral *Plexaura homomalla* [57,58,59]. In this organism, a bifunctional LOX-AOS enzyme was identified in which AOS forms the NH_2_-terminal portion and 8*R*-LOX the COOH-terminal portion of the polypeptide chain [59]. The two domains cooperate in oxylipin biosynthesis and catalyze consecutive steps in an eicosanoid-like biosynthetic pathway. Hereby, the 8*R*-LOX domain converts arachidonic acid to hydroperoxy-eicosatetraenoic acid (8*R*-HPETE), followed by transformation of 8*R*-HPETE to an allene oxide by the AOS domain. The product of the two coupled reactions is a likely precursor of marine prostanoid-like compounds, such as clavulones, which are cytotoxins implicated in the defense against microbial pests.

**Figure 2:**
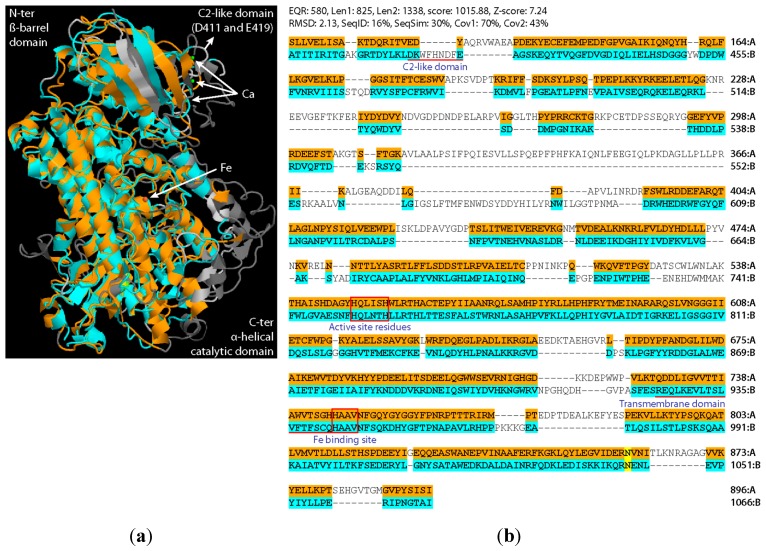
Structural models of the 8*R*-LOX of *Plexaura homomella* and LOX2 of Arabidopsis. A homology model of AtLOX2 was established with soybean LOX3 (PDB ID: 1LNH) as template [65] using SWISS-MODEL [66] and, in turn, super-positioned with the structure of the crystallized 8*R*-LOX domain (PDB ID: 2FNQ) of the soft coral 8*R*–LOX–AOS [59]. To overlay the structures, the Smith–Waterman superposition algorithm on the Calculate Structure Alignment.app was used. (**a**) 3D structure of LOX2 (mustard color) and the 8*R*-LOX domain (teal color) of the soft coral 8*R*–LOX–AOS. (**b**) Amino acid sequence alignment to highlight the conservation of structural domains and active site residues. Active site residues and iron binding sites (boxed) are indicated [62]. Transmembrane domain prediction was done using TMpred [67].

8*R*-LOX contains a NH_2_-terminal ß-barrel domain and a COOH-terminal, largely α-helical catalytic domain and is, thus, related to other lipoxygenases including LOX2 from Arabidopsis (Figure 2) [60,61,62], while the AOS of *P. homomalla* has sequence similarity to catalases, both in terms of the polypeptide fold and heme binding sites [5,6,60]. The NH_2_-terminal domain of 8*R*-LOX resembles the calcium-dependent membrane-binding module, termed C2 module, in phospholipases and kinases [63,64]. No such C2 domain is present in LOX2 of Arabidopsis (Figure 2). In the soft coral, 8*R*-LOX and AOS are linked by a short peptide, in the absence of which the two polypeptides adopt a LOX-AOS dimer conformation identical to that of the natural bifunctional enzyme [59]. Both in the linker-containing and linker-free states, the COOH-terminus of AOS was positioned close (10.5 Å and 13.5 Å) to the NH_2_-terminus of the 8*R*-LOX. Hereby, helix α2 of AOS established contacts with both the C2- and catalytic domains of 8*R*-LOX [59]. The intramolecular interface average diameter of ≈1000 Å was dominated by polar and charged amino acid interactions. Other studies revealed that helix α2 affects the conformations of active site ligands of both the 8*R*-LOX and AOS domains, allowing for communication between the two catalytic sites and controlling the flow of metabolites through the bifunctional enzyme [42].

**Figure 3:**
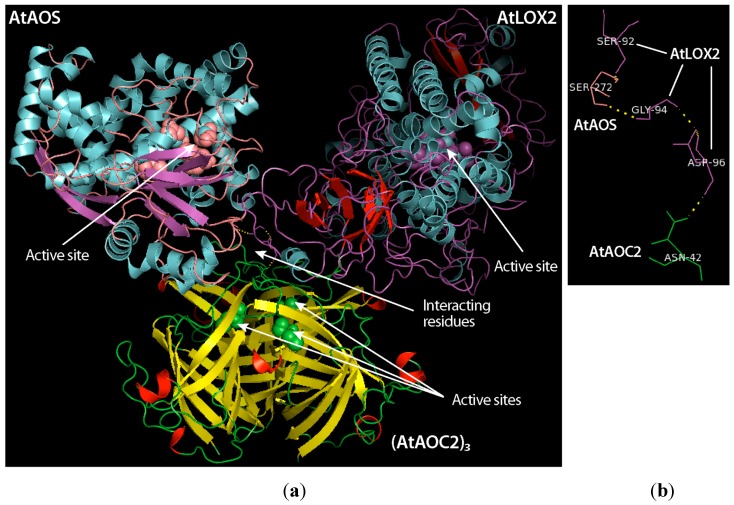
Structural model of the LOX2–AOS–AOC2 complex in chloroplasts. (**a**) Overall structure that was obtained by fitting the modelled LOX2 structures of Arabidopsis to the known X-ray structures of AOS [44] and AOC2 [32]. Amino acid residues suggested to be involved in catalysis of each enzyme as well as amino acid residues tentatively defined as residues mediating subunit interactions are indicated. (**b**) Cartoon highlighting the different amino acid pairs implicated in the different protein–protein interactions.

In Arabidopsis, LOX (LOX2) and AOS are not fused as in the soft coral but form a complex, which, in addition, contains AOC2. Crosslinking studies revealed that LOX2 interacts preferentially with AOS, whereas it does not seem to establish direct contacts with AOC2 being present as monomers and trimers. Modeling of the LOX2–AOS–AOC2 structure allowed proposing how these differential protein–protein interactions may be explained in molecular terms. Our top ranked model (Figure 3 and Table 1) suggests differential interactions between the three partner proteins, with unique amino acid residues of LOX2 interacting with unique amino acids of AOS and unique amino acid interactions between AOS and AOC2. Accordingly, Ser92 and Gly94 of LOX2 forming hydrogen bonds with Ser272 of AOS. Ser92 and Gly94 are localized in a loop region between β-sheets 1 and 2 of LOX2, presumably forming a surface-exposed region ready for binding AOS. On the other hand, Asp96 of LOX2 was found to potentially interact with Asn42 of AOC2. However, when the AOC2 homotrimer and AOS were modeled together in the absence of LOX2 (out of the ternary complex), Phe44 and Ser45 of AOC2 were found to interact with Ile65 and Pro64 of AOS, respectively. Interestingly, none of these amino acid interactions were seen in the modeled whole LOX2–AOS–AOC2 complex (Figure 3, Figure S1, and Table 1). 

A critical point in our model concerns the role of the lipid bilayers. Because the one trans-membrane (TM) domain predicted in LOX2 is part of the interaction site with AOS (Figure 3), we assume that LOX2 may need to sort out of the lipid bilayers in order to bind AOS.

Alternatively, some of the 5-LOX enzymes involved in leukotriene synthesis have been suggested to bind membranes via their catalytic domain [68,69]. Other reports on metazoan 15-LOX enzymes known to act as mediators of inflammation indicated an essential role of the catalytic domain for membrane localization and that catalytically driven membrane perforation provides a mechanism of programmed organelle degradation occurring during the differentiation of reticulocytes and maybe also in the clearance of eye lens cells [70,71]. For AOS, a single TM domain is predicted to be part of the active site as well. However, it seems likewise possible that some of the detergent-exposed hydrophobic α-helices, and particularly those designated α-F and α-H in the crystal structure of Lee et al. [44], could provide membrane anchors. In the case of the bifunctional enzyme from *P. homomalla*, the AOS domain, if expressed alone without the 8*R*-LOX domain, easily formed dimers via helix α2 [61]. However, because helix α2 also establishes part of the 8*R*–LOX–AOS interface, no dimerization of the full-length bifunctional enzyme was observed, as would be expected if helix α2 is present in a free state [45]. In case of the AOC trimer, one predictable TM domain is present per monomer, but it is also conceivable that some of the hydrophobic β-sheets forming the ß-barrel’s active site cavity in the trimer could mediate membrane binding and/or the interaction with AOS. In the case of the bifunctional 8*R*-LOX-AOS soft coral enzyme, the C2-module flanking the two catalytic domains mediates membrane binding and domain interaction in an either/or fashion [72]. How the supposed shuffling of enzymes between the lipid bilayers of the inner plastid envelope and the LOX2–AOS–AOC2 complex is regulated needs to be resolved in future work. 

## 4. Expression of *LOX2*, *AOS,* and *AOC* over Plant Development and Role of AOC Homo- and Hetero-Trimerization for Activity Regulation

**Figure 4:**
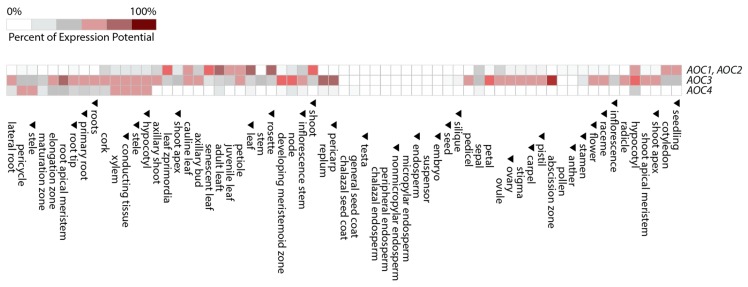
Tissue and development-specific expression of *AOC1*-*4* from *Arabidopsis thaliana*. The Genevestigator database [74] was used to contrast the specific expression pattern of *AOC1-4*. The patterns reveal a clear expression of *AOC1* and *2* in areal tissues, while being absent in roots and only very little expressed in flowers. *AOC4* is seemingly restricted to root tissues, whereas *AOC3* expression is detected at different levels in all tissues.

First glimpses suggestive of activity regulation through changing subunit compositions of the four different AOC isoenzymes in the homo- and heterotrimers of the LOX2–AOS–(AOC)_3_ complex have been provided by Otto and co-workers [73]. BiFC studies did not reveal structural constraints for AOC homo- and hetero-trimerization. The analysis of publicly available transcriptomics data deposited in the Genevestigator database nevertheless indicates non-overlapping functions of *AOC1* and *2* versus *AOC3* and *4* in Arabidopsis. In fact, *AOC1* and *AOC2* are expressed in different organs and at other stages of development than *AOC3* and *AOC4* (Figure 4).

**Figure 5:**
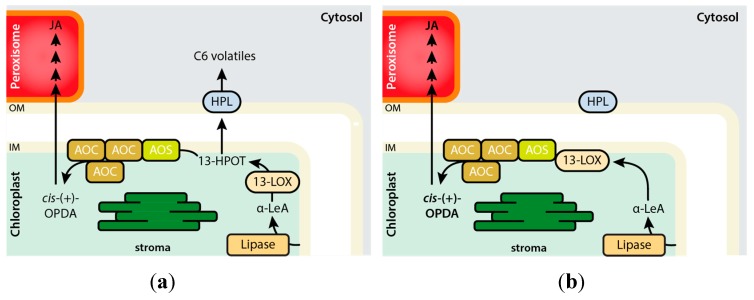
Compartmentalization of the AOS and HPL branches of the Vick and Zimmerman pathway in plants. (**a**) Under unperturbed conditions, the link between LOX2 and AOS is less strong, causing a portion of 13-HPOT to leak out of the enzymatic channel. The free 13-HPOT serves as substrate for HPL to produce green leaf volatiles. (**b**) In response to herbivory, however, the amount of chloroplastic AOS and AOC increases, effectively recruiting LOX2 into the LOX2–AOS–AOC complex. This prevents leakage of 13-HPOT and consequently promotes *cis*-(+)-OPDA production.

Because bioinformatics approaches revealed the non-conservation of amino acid residues that were tentatively identified as putative interaction sites with AOS, we conclude that AOC1 and AOC2, being present as homo- or hetero-trimers, are the basic components of the discovered oxylipin biosynthesis complex in the chloroplast. Due to its unique composition and location in chloroplasts, the LOX2–AOS–AOC1/2 complex assures oxylipin precursor biosynthesis, obviously avoiding the competing side reactions such as those catalyzed by HPL. As a consequence, plants can tightly control the quantity of JA over plant development and maintain or boost JA production in response to stress rapidly. By contrast, the expression of HPL appears to remain low under most developmental conditions and augments only when plants are challenged by feeding insects. Whether under these circumstances HPL then competes with AOS for 13-HPOT has not been established. It might well be that those other LOX enzymes present at different locations in the plant cell could provide the substrate for HPL-driven aldehyde synthesis (Figure 5; see also [30,35]). The localization of HPL at the outer surface of chloroplasts could favor such action but would require either a release of the membrane-bound enzyme or collapse of intracellular compartmentalization and access of HPL to non-chloroplastic membrane lipids. Further work is needed to address this hypothesis.

## 5. Conclusions and Perspectives

**Figure 6:**
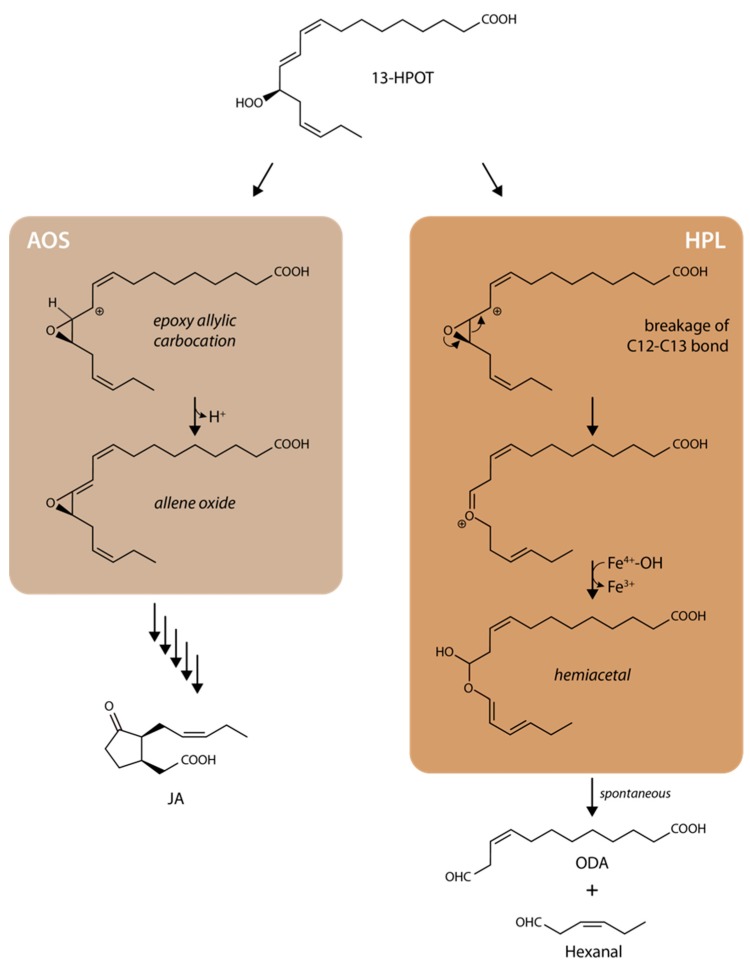
Reaction mechanism proposed on the basis of the bifunctional 8*R*–LOX–AOS and 8*R*–LOX–HPL enzymes from cyanobacteria and *Capsella imbricate*. Modified after Teder et al. [79].

AOS and HPL are non-canonical CYP74 enzymes that may have their origin in the last common ancestor of plants and animals while being absent from most metazoan lineages known to date [44]. AOS and HPL obviously evolved by precluding mono-oxygenation chemistry. As shown by Lee et al. [44], their molecular structures are remarkably similar. However, subtle differences in substrate positioning within the active site permits to exquisitely control the reactivity of catalytic intermediates formed from a common substrate for achieving product specificity. Using a mutagenesis approach, Lee et al. [44] were able to convert Arabidopsis AOS into an HPL-like enzyme. The mutational changes concerned just two amino acid residues but were sufficient to control substrate entrance and positioning, permitting alternative reactions by AOS and HPL and, thus, promoting either JA precursor biosynthesis or green volatile production, respectively. Bioinformatics studies showed that although the eudicot (*Arabidopsis thaliana*) and monocot (*Oryza sativa*) branches of angiosperms diverged 140–180 million years ago [75], introduction of F92L substitution into one of the two AOS genes found in rice was apparently sufficient to alter product specificity and establish the HPL branch [44]. AOS, as discussed here, is capable of interacting with two other entry enzymes of the oxylipin pathway, that is, LOX2 and AOC2, and thereby establishing a complex involved in boosting JA production [53]. Modeling the structure of the discovered complex revealed several unique features as compared to the bifunctional 8*R*-LOX-AOS enzyme in soft coral. While 8*R*-LOX is structurally similar to plant LOX2, its AOS domain is related to catalases [58,59,61]. Fusion proteins equivalent/corresponding to that described for the soft coral *Plexaura homomella* operate in cyanobacteria such as *Anabena* and *Nostoc* sp. PCC 7120 [76,77] and other soft corals [78]. Similar to the soft coral enzymes but in marked contrast to the enzymes from higher plants, the catalase domain of the cyanobacterial enzymes converted fatty acid hydroperoxides that are in the *R* configuration into their corresponding products. By analogy to what was recently described for the 8*R*-LOX-AOS from *Capnella imbricate* [79], the transformation of the linolenate hydroperoxide to the allylic epoxides is likely to involve a carbocation intermediate [76,80] (Figure 6).

Conversion of conjugated diene hydroperoxides to allyl carbocations, thus, occurs in virtually the same way as in the enzymatic synthesis of allene oxides by plant AOS and in the transformations of prostaglandin endoperoxides to prostacyclin and thromboxane A2 in metazoans [81,82]. Of the two catalase-lipoxygenase fusion protein genes identified in the coral *Capnella imbricate*, gene A encodes a wound-responsive AOS-type enzyme and gene B encodes an HPL-type enzyme specialized for the synthesis of short-chain aldehydes [78,83]. As demonstrated by site-directed mutagenesis, defined and quite limited changes in active site amino acid residues, such as those encountered in the engineered F150L and YS176-177NL HPL derivatives, shifted the reaction specificity from HPL to AOS [79]. Together, these findings put new fuel into the discussion on the origin of JA and green leaf volatile synthesis in nature. This finding provides strong evidence for an independent, second origin of AOS and suggests a case of convergent evolution. The reasons of these apparently different mechanisms to control the flow of metabolites in plants and soft corals remain to be established.

## Figures and Tables

**Table 1:** Amino acids identified to be potentially implicated in LOX2–AOS–AOC2 complex formation.

Amino Acid Interactions Observed in the Ternary Complex	Amino Acid Interactions Observed in an Arbitrary AOS–AOC2 Complex
LOX2-Ser92–AOS-Ser272	AOS-Pro64–AOC2-Ser45
LOX2-Gly94–AOS-Ser272 LOX2-Asp96–AOC2-Asn42	AOS-Ile65–AOC2-Phe44

## Supplementary Materials

The following are available online at https://www.mdpi.com/1422-0067/20/12/3064/s1.

## Abbreviations

13-HPOT	(9*Z*11*E*15*Z*13*S*)-13-hydroperoxy-9,11,15-octadecatrienoic acid
α-LeA	α-linolenic acid
AOC	Allene oxide cyclase
AOS	Allene oxide synthase
CYP	Cytochrome P450 enzymes
EOT	12,13(*S*)-epoxy-9(*Z*),11,15(*Z*)-octadecatrienoic acid
HPL	Hydroperoxide lyase
JA	Jasmonic acid
LOX	Lipoxygenase
ODA	12-oxo-*cis*-9-dodecenoic acid
OPDA	*cis*-(+)-12-oxophytodienoic acid

## References

[1] Peters-Golden M., Brock T.G. (2000). Intracellular compartmentalization of leukotriene biosynthesis. Am. J. Respir. Crit. Care Med..

[2] Soberman R.J., Christmas P. (2003). The organization and consequences of eicosanoid signaling. J. Clin. Investig..

[3] Sharma J.N., Mohammed L.A. (2006). The role of leukotrienes in the pathophysiology of inflammatory disorders: Is there a case for revisiting leukotrienes as therapeutic targets?. Inflammopharmacology.

[4] Corey E.J., D’Alarcao M., Matsuda S.P.T., Lansbury P.T., Yamada Y. (1987). Intermediacy of 8-(*R*)-HPETE in the conversion of arachidonic acid to pre-clavulone a by *Clavularia viridis*. Implications for the biosynthesis of marine prostanoids. J. Am. Chem. Soc..

[5] Corey E.J., Matsuda S.P.T., Nagata R., Cleaver M.B. (1988). Biosynthesis of 8-*R*-HPETE and preclavulone-A from arachidonate in several species of caribbean coral. A widespread route to marine prostanoids. Tetrahedron Lett..

[6] Tijet N., Brash A.R. (2002). Allene oxide synthases and allene oxides. Prostaglandins Other Lipid Medi..

[7] Wasternack C., Strnad M. (2018). Jasmonates: News on occurrence, biosynthesis, metabolism and action of an ancient group of signaling compounds. Int. J. Mol. Sci..

[8] Reinbothe C., Springer A., Samol I., Reinbothe S. (2009). Plant oxylipins: Role of jasmonic acid during programmed cell death, defence and leaf senescence. FEBS J..

[9] Yan Y., Borrego E., Kolomiets M.V., Valenzuela Baez R.E. (2013). Jasmonate biosynthesis, perception and function in plant development and stress responses. Lipid Metabolism.

[10] Wasternack C., Feussner I. (2018). The oxylipin pathways: Biochemistry and function. Annu. Rev. Plant Biol..

[11] Schommer C., Palatnik J.F., Aggarwal P., Chetelat A., Cubas P., Farmer E.E., Nath U., Weigel D. (2008). Control of jasmonate biosynthesis and senescence by miR319 targets. PLoS Biol.

[12] Hoffmann M., Hentrich M., Pollmann S. (2011). Auxin-oxylipin crosstalk: Relationship of antagonists. J. Integr. Plant Biol..

[13] Gfeller A., Liechti R., Farmer E.E. (2010). Arabidopsis jasmonate signaling pathway. Sci. Signal.

[14] Farmer E.E., Ryan C.A. (1990). Interplant communication: Airborne methyl jasmonate induces synthesis of proteinase inhibitors in plant leaves. Proc. Natl. Acad. Sci. USA.

[15] Mattiacci L., Dicke M., Posthumus M.A. (1995). ß-Glucosidase: An elicitor of herbivore-induced plant odor that attracts host-searching parasitic wasps. Proc. Natl. Acad. Sci. USA.

[16] Baldwin I.T. (1999). Functional interactions in the use of direct and indirect defences in native Nicotiana plants. Novartis Found Symp..

[17] Kessler A., Baldwin I.T. (2001). Defensive function of herbivore-induced plant volatile emissions in nature. Science.

[18] Wu J., Wang L., Baldwin I.T. (2008). Methyl jasmonate-elicited herbivore resistance: Does MeJA function as a signal without being hydrolyzed to JA?. Planta.

[19] Arimura G., Matsui K., Takabayashi J. (2009). Chemical and molecular ecology of herbivore-induced plant volatiles: Proximate factors and their ultimate functions. Plant Cell Physiol..

[20] Arimura G., Shiojiri K., Karban R. (2010). Acquired immunity to herbivory and allelopathy caused by airborne plant emissions. Phytochemistry.

[21] Wu J., Baldwin I.T. (2010). New insights into plant responses to the attack from insect herbivores. Annu. Rev. Genet..

[22] ul Hassan M.N., Zainal Z., Ismail I. (2015). Green leaf volatiles: Biosynthesis, biological functions and their applications in biotechnology. Plant Biotechnol. J..

[23] Matsui K., Koeduka T., Nakamura Y., Li-Beisson Y. (2016). Green leaf volatiles in plant signaling and response. Lipids in Plant and Algae Development, Subcellular Biochemistry.

[24] Ameye M., Allmann S., Verwaeren J., Smagghe G., Haesaert G., Schuurink R.C., Audenaert K. (2018). Green leaf volatile production by plants: A meta-analysis. New Phytol..

[25] Schaller A., Stintzi A. (2009). Enzymes in jasmonate biosynthesis*—*Structure, function, regulation. Phytochemistry.

[26] Gfeller A., Dubugnon L., Liechti R., Farmer E.E. (2010). Jasmonate biochemical pathway. Sci. Signal.

[27] Griffiths G. (2015). Biosynthesis and analysis of plant oxylipins. Free Radic. Res..

[28] Vick B.A., Zimmerman D.C. (1984). Biosynthesis of jasmonic acid by several plant species. Plant Physiol..

[29] Vick B.A., Zimmerman D.C. (1987). Pathways of fatty acid hydroperoxide metabolism in spinach leaf chloroplasts. Plant Physiol..

[30] Feussner I., Wasternack C. (2002). The lipoxygenase pathway. Annu. Rev. Plant Biol..

[31] Laudert D., Pfannschmidt U., Lottspeich F., Holländer-Czytko H., Weiler E.W. (1996). Cloning, molecular and functional characterization of *Arabidopsis thaliana* allene oxide synthase (CYP 74), the first enzyme of the octadecanoid pathway to jasmonates. Plant Mol. Biol..

[32] Hofmann E., Zerbe P., Schaller F. (2006). The crystal structure of *Arabidopsis thaliana* allene oxide cyclase: Insights into the oxylipin cyclization reaction. Plant Cell.

[33] Schaller F., Zerbe P., Reinbothe S., Reinbothe C., Hofmann E., Pollmann S. (2008). The allene oxide cyclase family of *Arabidopsis thaliana*: Localization and cyclization. FEBS J..

[34] Hofmann E., Pollmann S. (2008). Molecular mechanism of enzymatic allene oxide cyclization in plants. Plant Physiol. Biochem..

[35] Joo Y.C., Oh D.K. (2012). Lipoxygenases: Potential starting biocatalysts for the synthesis of signaling compounds. Biotechnol. Adv..

[36] Croft K., Jüttner F., Slusarenko A.J. (1993). Volatile Products of the Lipoxygenase Pathway Evolved from *Phaseolus vulgaris* (L.) Leaves Inoculated with *Pseudomonas syringae* pv *phaseolicola*. Plant Physiol..

[37] Bate N.J., Rothstein S.J. (1998). C6-volatiles derived from the lipoxygenase pathway induce a subset of defense-related genes. Plant J..

[38] Blée E. (1998). Phytooxylipins and plant defense reactions. Prog. Lipid Res..

[39] Böttcher C., Pollmann S. (2009). Plant oxylipins: Plant responses to 12-oxo-phytodienoic acid are governed by its specific structural and functional properties. FEBS J..

[40] The Arabidopsis Genome Initiative (2000). Analysis of the genome sequence of the flowering plant *Arabidopsis thaliana*. Nature.

[41] Song W.C., Brash A.R. (1991). Purification of an allene oxide synthase and identification of the enzyme as a cytochrome P-450. Science.

[42] Froehlich J.E., Itoh A., Howe G.A. (2001). Tomato allene oxide synthase and fatty acid hydroperoxide lyase, two cytochrome P450s involved in oxylipin metabolism, are targeted to different membranes of chloroplast envelope. Plant Physiol..

[43] La Camera S., Gouzerh G., Dhondt S., Hoffmann L., Fritig B., Legrand M., Heitz T. (2004). Metabolic reprogramming in plant innate immunity: The contributions of phenylpropanoid and oxylipin pathways. Immunol. Rev..

[44] Lee D.S., Nioche P., Hamberg M., Raman C.S. (2008). Structural insights into the evolutionary paths of oxylipin biosynthetic enzymes. Nature.

[45] Li L., Chang Z., Pan Z., Fu Z.Q., Wang X. (2008). Modes of heme binding and substrate access for cytochrome P450 CYP74A revealed by crystal structures of allene oxide synthase. Proc. Natl. Acad. Sci. USA.

[46] Williams P.A., Cosme J., Sridhar V., Johnson E.F., McRee D.E. (2000). Microsomal cytochrome P450 2C5: Comparison to microbial P450s and unique features. J. Inorg. Biochem..

[47] Tyagi C., Singh A., Singh I.K. (2016). Mechanistic insights into mode of action of rice allene oxide synthase on hydroxyperoxides: An intermediate step in herbivory-induced jasmonate pathway. Comput. Biol. Chem..

[48] Williams P.A., Cosme J., Sridhar V., Johnson E.F., McRee D.E. (2000). Mammalian microsomal cytochrome P450 monooxygenase: Structural adaptations for membrane binding and functional diversity. Mol. Cell.

[49] Nelson D.R. (2006). Plant cytochrome P450s from moss to poplar. Phytochem. Rev..

[50] Grechkin A.N., Hamberg M. (2004). The “heterolytic hydroperoxide lyase” is an isomerase producing a short-lived fatty acid hemiacetal. Biochim. Biophys. Acta.

[51] Hooper C.M., Castleden I.R., Tanz S.K., Aryamanesh N., Millar A.H. (2017). SUBA4: The interactive data analysis centre for Arabidopsis subcellular protein locations. Nucleic Acids Res..

[52] Froehlich J.E., Wilkerson C.G., Ray W.K., McAndrew R.S., Osteryoung K.W., Gage D.A., Phinney B.S. (2003). Proteomic study of the *Arabidopsis thaliana* chloroplastic envelope membrane utilizing alternatives to traditional two-dimensional electrophoresis. J. Proteome Res..

[53] Pollmann S., Springer A., Rustgi S., Wettstein D.V., Kang C., Reinbothe C., Reinbothe S. (2019). Substrate channeling in oxylipin biosynthesis through a protein complex in the plastid envelope of *Arabidopsis thaliana*. J. Exp. Bot..

[54] Emanuelsson O., Nielsen H., von Heijne G. (1999). ChloroP, a neural network-based method for predicting chloroplast transit peptides and their cleavage sites. Protein Sci..

[55] Emanuelsson O., Nielsen H., Brunak S., von Heijne G. (2000). Predicting subcellular localization of proteins based on their N-terminal amino acid sequence. J. Mol. Biol..

[56] Farmaki T., Sanmartin M., Jimenez P., Paneque M., Sanz C., Vancanneyt G., Leon J., Sanchez-Serrano J.J. (2007). Differential distribution of the lipoxygenase pathway enzymes within potato chloroplasts. J. Exp. Bot..

[57] Corey E.J., Washburn W.N., Chen J.C. (1973). Prostaglandin A2 synthetase complex from *Plexaura homomalla*. J. Am. Chem. Soc..

[58] Koljak R., Boutaud O., Shieh B.H., Samel N., Brash A.R. (1997). Identification of a naturally occurring peroxidase-lipoxygenase fusion protein. Science.

[59] Gilbert N.C., Niebuhr M., Tsuruta H., Bordelon T., Ridderbusch O., Dassey A., Brash A.R., Bartlett S.G., Newcomer M.E. (2008). A covalent linker allows for membrane targeting of an oxylipin biosynthetic complex. Biochemistry.

[60] Oldham M.L., Brash A.R., Newcomer M.E. (2005). Insights from the X-ray crystal structure of coral 8R-lipoxygenase: Calcium activation via a C2-like domain and a structural basis of product chirality. J. Biol. Chem..

[61] Oldham M.L., Brash A.R., Newcomer M.E. (2005). The structure of coral allene oxide synthase reveals a catalase adapted for metabolism of a fatty acid hydroperoxide. Proc. Natl. Acad. Sci. USA.

[62] Youn B., Sellhorn G.E., Mirchel R.J., Gaffney B.J., Grimes H.D., Kang C. (2006). Crystal structures of vegetative soybean lipoxygenase VLX-B and VLX-D, and comparisons with seed isoforms LOX-1 and LOX-3. Proteins Struct. Funct. Bioinform..

[63] Rizo J., Südhof T.C. (1998). C2-domains, structure and function of a universal Ca^2+^-binding domain. J. Biol. Chem..

[64] Cho W., Stahelin R.V. (2006). Membrane binding and subcellular targeting of C2 domains. Biochim. Biophys. Acta.

[65] Skrzypczak-Jankun E., Amzel L.M., Kroa B.A., Funk M.O. (1997). Structure of soybean lipoxygenase L3 and a comparison with its L1 isoenzyme. Proteins.

[66] Schwede T., Kopp J., Guex N., Peitsch M.C. (2003). SWISS-MODEL: An automated protein homology-modeling server. Nucleic Acids Res..

[67] Hofmann K., Stoffel W. (1993). Tmbase—A database of membrane spanning proteins segments. Biol. Chem. Hoppe-Seyler.

[68] Brock T.G., Healy A.M. (2000). Nuclear import of arachidonate 5-lipoxygenase. Arch. Immunol. Ther. Exp..

[69] Chen X.S., Funk C.D. (2001). The N-terminal “beta-barrel” domain of 5-lipoxygenase is essential for nuclear membrane translocation. J. Biol. Chem..

[70] Schewe T., Rapoport S.M., Kuhn H. (1986). Enzymology and physiology of reticulocyte lipoxygenase: Comparison with other lipoxygenases. Adv. Enzymol. Relat. Areas Mol. Biol..

[71] van Leyen K., Duvoisin R.M., Engelhardt H., Wiedmann M. (1998). A function for lipoxygenase in programmed organelle degradation. Nature.

[72] Cook A.C., Ho C., Kershner J.L., Malinowski S.A., Moldveen H., Stagliano B.A., Slater S.J. (2006). Competitive binding of protein kinase Calpha to membranes and Rho GTPases. Biochemistry.

[73] Otto M., Naumann C., Brandt W., Wasternack C., Hause B. (2016). Activity Regulation by heteromerization of Arabidopsis allene oxide cyclase family members. Plants.

[74] Hruz T., Laule O., Szabo G., Wessendorp F., Bleuler S., Oertle L., Widmayer P., Gruissem W., Zimmermann P. (2008). Genevestigator v3: A reference expression database for the meta-analysis of transcriptomes. Adv. Bioinform..

[75] Bell C.D., Soltis D.E., Soltis P.S. (2005). The age of the angiosperms: A molecular timescale without a clock. Evolution.

[76] Schneider C., Niisuke K., Boeglin W.E., Voehler M., Stec D.F., Porter N.A., Brash A.R. (2007). Enzymatic synthesis of a bicyclobutane fatty acid by a hemoprotein lipoxygenase fusion protein from the cyanobacterium *Anabaena* PCC 7120. Proc. Natl. Acad. Sci. USA.

[77] Lang I., Gobel C., Porzel A., Heilmann I., Feussner I. (2008). A lipoxygenase with linoleate diol synthase activity from *Nostoc sp.*. PCC 7120. Biochem. J..

[78] Teder T., Lõhelaid H., Boeglin W.E., Calcutt W.M., Brash A.R., Samel N. (2015). A Catalase-related hemoprotein in coral is specialized for synthesis of short-chain aldehydes: Discovery of P450-type hydroperoxide lyase activity in a catalase. J. Biol. Chem..

[79] Teder T., Lõhelaid H., Samel N. (2017). Structural and functional insights into the reaction specificity of catalase-related hydroperoxide lyase: A shift from lyase activity to allene oxide synthase by site-directed mutagenesis. PLoS ONE.

[80] Niisuke K., Boeglin W.E., Murray J.J., Schneider C., Brash A.R. (2009). Biosynthesis of a linoleic acid allylic epoxide: Mechanistic comparison with its chemical synthesis and leukotriene A biosynthesis. J. Lipid Res..

[81] Hecker M., Ullrich V. (1989). On the mechanism of prostacyclin and thromboxane A2 biosynthesis. J. Biol. Chem..

[82] Gerwick W.H. (1996). Epoxy allylic carbocations as conceptual intermediates in the biogenesis of diverse marine oxylipins. Lipids.

[83] Lõhelaid H., Teder T., Tõldsepp K., Ekins M., Samel N. (2014). Up-regulated expression of AOS-LOXa and increased eicosanoid synthesis in response to coral wounding. PLoS ONE.

