# Supplementary material for: ALLENE OXIDE SYNTHASE and HYDROPEROXIDE LYASE, Two Non-Canonical Cytochrome P450s in Arabidopsis thaliana and Their Different Roles in Plant Defense
Source: Int J Mol Sci. 2019 Jun 23;20(12):3064. doi: 10.3390/ijms20123064 (PMC6627107; doi:10.3390/ijms20123064)
Supplement: Supplementary file 1 [file ijms-20-03064-s001.zip › Figure S1.docx]

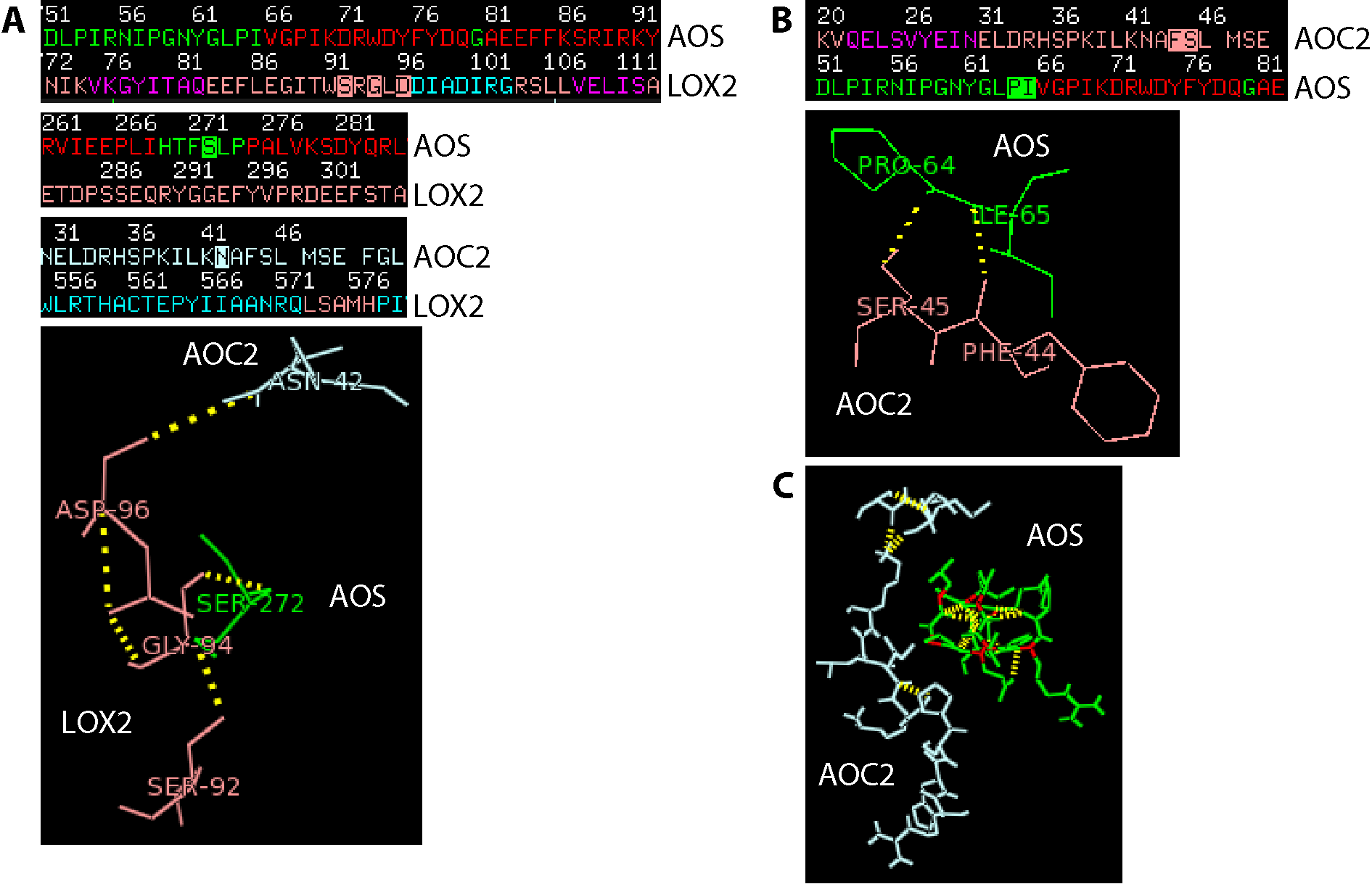


Figure S1. Protein-protein docking analysis of the LOX2-AOS-AOC2 complex in chloroplasts. The presented structures were obtained by fitting the modelled LOX2 structure of Arabidopsis to the know X-ray structures of AOS [43] and AOC2 [32]. (**A**) Primary amino acid structure (top panel) and cartoon (lower panel) depicting the putative amino acid residues (highlighted) involved in subunit interactions between AOS and LOX2 as well as AOC2 and LOX2. (**B**) Amino acid sequence (top panel) and cartoon (middle panel) showing the amino acid residues (highlighted) tentatively involved in the interaction between AOS and AOC2. (**C**) Remarkably, the calculated interactions could not be observed in the modelled ternary complex.
